# Overexpression of a Cotton Aquaporin Gene *GhTIP1;1-like* Confers Cold Tolerance in Transgenic *Arabidopsis*

**DOI:** 10.3390/ijms23031361

**Published:** 2022-01-25

**Authors:** Gongmin Cheng, Mengdi Wang, Longyan Zhang, Hengling Wei, Hantao Wang, Jianhua Lu, Shuxun Yu

**Affiliations:** 1 State Key Laboratory of Cotton Biology, Institute of Cotton Research of CAAS, Anyang 455000, China; cgm900923@163.com (G.C.); 15614991132@163.com (L.Z.); henglingwei@163.com (H.W.); w.wanghantao@163.com (H.W.); lujh212760@163.com (J.L.); 2 School of Biological Science and Food Engineering, Chuzhou University, Chuzhou 239000, China; wmd951101@163.com; 3 College of Agronomy, Northwest A&F University, Yangling, Xianyang 712100, China; 4 School of Life Science, Northeast Normal University, Changchun 130024, China; 5 College of Agronomy, Hebei Agricultural University, Baoding 071001, China

**Keywords:** *Gossypium hirsutum*, *GhTIP1;1-like*, cold tolerance, virus-induced gene silencing (VIGS), overexpression, *Arabidopsis thaliana*

## Abstract

Cold stress can significantly affect the development, yield, and quality of crops and restrict the geographical distribution and growing seasons of plants. Aquaporins are the main channels for water transport in plant cells. Abiotic stresses such as cold and drought dehydrate cells by changing the water potential. In this study, we cloned a gene *GhTIP1;1-like* encodes tonoplast aquaporin from the transcriptome database of cotton seedlings after cold stress. Expression analysis showed that *GhTIP1;1-like* not only responds to cold stress but was also induced by heat, drought and salt stress. Subcellular localization showed that the protein was anchored to the vacuole membrane. Promoter deletion analysis revealed that a MYC motif within the promoter region of *GhTIP1;1-like* were the core *cis*-elements in response to low temperature. Virus-induced gene silencing (VIGS) and histochemical staining indicate that *GhTIP1;1-like* plays a positive role in plant cold tolerance. Overexpression of *GhTIP1;1-like* in *Arabidopsis* delayed the senescence process and enhanced the cold tolerance of transgenic plants. Compared with the wild type, the soluble protein concentration and peroxidase activity of the transgenic lines under cold stress were higher, while the malondialdehyde content was lower. In addition, the expression levels of cold-responsive genes were significantly increased in transgenic plants under cold stress. Our results indicate that *GhTIP1;1-like* could respond to different abiotic stresses and be positively involved in regulating the cold tolerance of cotton.

## 1. Introduction

As immobile organisms, plants should adjust their water balance to cope with adverse environmental challenges such as low temperature, water deficit and salt stress [1]. In plants, water molecules are transported across membranes through aquaporins (AQPs). AQPs belong to the superfamily of major intrinsic proteins (MIPs) that promote the crossing of water molecules and uncharged solutes across cell membranes. According to previous studies, 35, 33 and 32 AQP genes have been identified in *Arabidopsis*, rice and maize, respectively [2,3,4]. Most AQPs can be divided into four categories based on sequence homology: plasma membrane intrinsic proteins (PIPs), tonoplast intrinsic proteins (TIPs), nodulin26-like intrinsic proteins (NIPs) and small basic intrinsic proteins (SIPs). In plants, TIPs were the first aquaporins reported, including α-, β-, γ-, δ- and ε- subunits, which have high sequence homology [5]. TIPs have been found to have a strong water transport capacity, which can be up to 100-fold that of PIP in *Xenopus* oocytes [6].

TIPs are multifunctional channel proteins that not only transport water but also many other small solutes, such as NH_3_, urea, H_2_O_2_, and glycerol [7]. It has been found that TIPs have a strong water transport capacity, which can be up to 100-fold than that of PIP in *Xenopus oocytes* [6]. In plant, cells have to adjust their water homeostasis in response to adverse conditions such as drought, salinity, and cold [1]. Furthermore, plant water homeostasis can be disturbed by these environmental stimuli due to the decline of water potential in the cellular or extracellular environment that affects water absorption and loss of plant cells. It has been revealed that the expression levels of both *TIP*s and *PIP*s could be regulated by different environmental stimuli [8,9].

TIPs have been shown to play an important role in response to a variety of abiotic stresses, such as drought, salinity and oxidative stress [10]. Double mutant *tip1;1 tip1;2* of *Arabidopsis* showed increased anthocyanin content, decreased catalase activity and vigorous growth [11]. The number of lateral roots of *Arabidopsis* triple mutant *tip1;1 tip1;2 tip2;1* was significantly reduced, and the development of lateral roots was adversely affected [12]. In addition, the *TIP1;1* RNAi plants showed dwarf symptoms, early senescence and lesion formation, and the starch and apoplastic carbohydrate contents were significantly increased in *TIP1;1* RNAi plants [13]. In contrast, overexpression of the *TIP* gene can positively affect plant development and stress resistance. It was reported that overexpressed of ginseng *PgTIP1;1* gene in *Arabidopsis* accelerated plant growth, enhanced salt tolerance, and changed drought tolerance and cold adaptability [14]. Moreover, overexpression of *PgTIP1;1* also enhanced water absorption and evaporation rate and increased seed size and quantity [15].

In our previous study, a cold-responsive gene encoding tonoplast aquaporin was cloned from the cotyledons of upland cotton (*Gossypium hirsutum* L.) and named *GhTIP1;1-like* [16]. The *GhTIP1;1-like* gene is homologous to the tonoplast aquaporin gene *GhTIP1;1* cloned from cotton cotyledons in a previous study [17]. It has been revealed that overexpression of the *GhTIP1;1* gene in yeast significantly enhanced freezing resistance and improved survival rate at low temperatures. Sequence analysis revealed that the *GhTIP1;1-like* gene was reported by a previous study (GenBank accession: BK007054), but little is known about its function [18]. To further characterize the function of *GhTIP1;1-like* in plants, we investigated the phenotypes of *GhTIP1;1-like* VIGS cotton or transgenic *Arabidopsis* plants in cold. The results showed that the *GhTIP1;1-like* gene could respond to various abiotic stresses such as cold, heat, drought and salt, and the virus-induced silencing led to the weakening of cold tolerance of cotton plants. We also found revealed that the fragment from −33 bp to −1033 bp containing an MYC *cis*-element was one of the critical motifs responding to chilling in the *GhTIP1;1-like* promoter. Moreover, overexpression of *GhTIP1;1-like* in *Arabidopsis* resulted in delayed senescence, longer fruit pods and more seeds per pod, and significantly enhanced cold tolerance of transgenic lines.

## 2. Results

### 2.1. Identification and Characterization of GhTIP1;1-like

**Figure 1:**
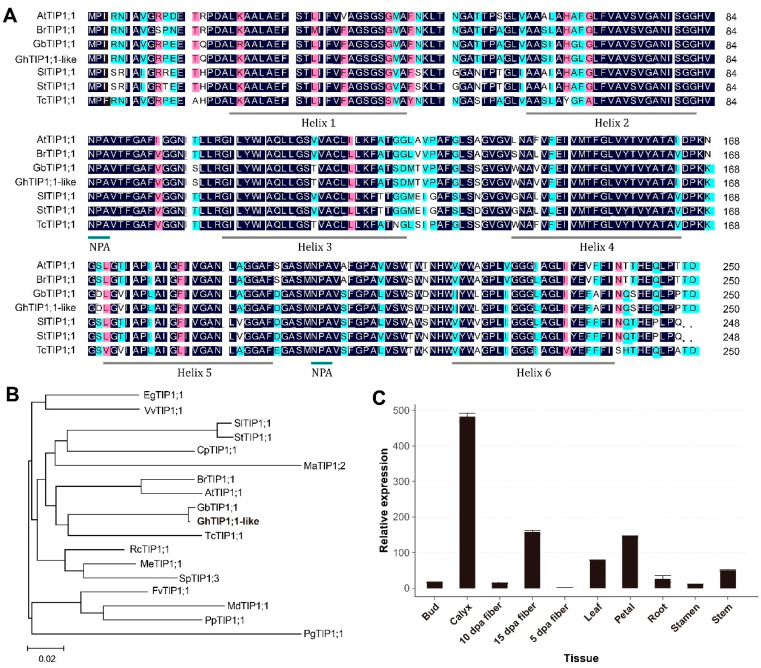
Sequence and expression pattern analysis of *GhTIP1;1-like*. (**A**) Amino acid sequence alignment between GhTIP1;1-like and other TIP1;1 proteins. (**B**) Phylogenetic analysis of GhTIP1;1-like and proteins from different plant species. The phylogenetic tree was constructed by the neighbour-joining (NJ) method using MEGA 6.0 software and the TIP1;1 protein sequences of *Arabidopsis thaliana* (Accession No. OAP07978.1), *Panax ginseng* (Accession No. DQ23728), *Brassica rapa* (Accession No. XP_009141610.1), *Theobroma cacao* (Accession No. XP_007027468.1), *Carica papaya* (Accession No. XP_021893349.1), *Eucalyptus grandis* (Accession No. XP_010037134.1), *Fragaria vesca* (Accession No. XP_004304478.1), *Gossypium barbadense* (Accession No. PPD81648.1), *Malus domestica* (Accession No. XP_008387528.2), *Solanum lycopersicum* (Accession No. XP_004249473.1), *Manihot esculenta* (Accession No. XP_021620289.1), *Musa acuminata* (Accession No. XP_009421351.1), *Prunus persica* (Accession No. XP_007202475.1), *Ricinus communis* (Accession No. EEF42870.1), *Salix purpurea* (Accession No. ALK82469.1), *Solanum tuberosum* (Accession No. XP_006339093.1), *Vitis vinifera* (Accession No. RVX11762.1). (**C**) The expression patterns of *GhTIP1;1-like* in different cotton organs. Data are shown as the mean ± SE (*n* = 3).

In a previous study, we identified a cold-induced AQP gene homologous to *AtTIP1;1* and named it *GhTIP1;1-like*. The full-length open reading frame (ORF) of *GhTIP1;1-like* gene was isolated from TM-1 cotyledons after cold treatment. The *GhTIP1;1-like* gene encodes a protein of 251 amino acid residues with a molecular weight of 25.89 kD and theoretical pI of 4.95 (Table S1). The GhTIP1;1-like protein sequence was composed of 20 amino acid residues, of which Ala (13.55%), Gly (13.15%), Leu (10.36%) and Val (8.76%) were the most abundant. Protein sequence alignment found that GhTIP1;1-like has two NPA domains and six hydrophobic regions (α-helices), which are typical characteristics of tonoplast aquaporins (Figure 1A). Furthermore, phylogenetic tree was constructed, which revealed that GhTIP1;1-like shared the same subgroup with GbTIP1;1 (PPD81648.1, from Gossypium barbadense) and TcTIP1;1 (XP_007027468.1, from Theobroma cacao), and most closely related to GbTIP1;1 (Figure 1B).

### 2.2. Expression Analysis of GhTIP1;1-like in G. hirsutum

To analyze the expression of *GhTIP1;1-like* in cotton, RT-qPCR was used to detect the expression level of *GhTIP1;1-like* in different tissues of TM-1. We found that *GhTIP1;1-like* has different expression levels in different tissues of cotton (Figure 1C). The expression level in cotton sepals was the highest, followed by the expression level in 15 dpa fibers, petals, leaves, stems and roots. However, the expression level in 10 dpa fibers and stamens was relatively low, and the lowest was in 5 dpa fibers.

### 2.3. Subcellular Localization of GhTIP1;1-like

**Figure 2:**
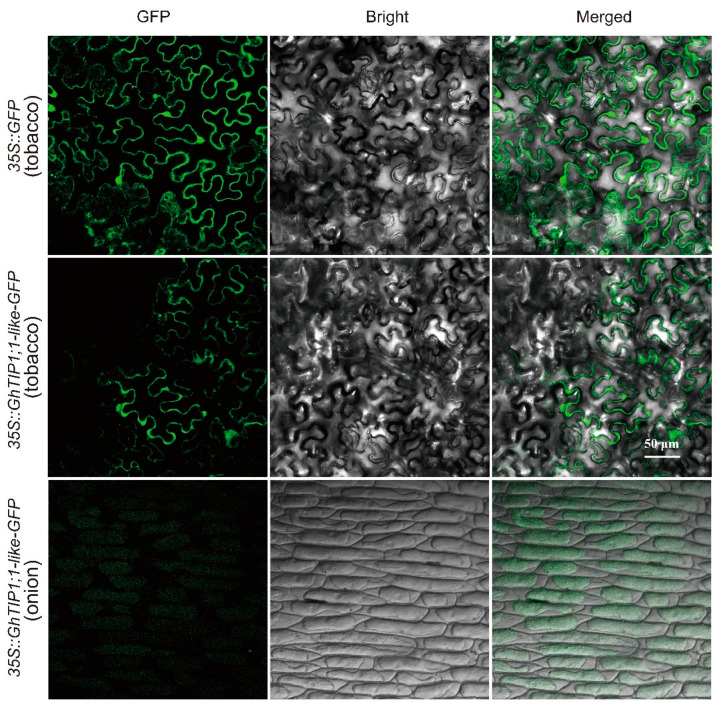
Subcellular localization of *GhTIP1;1-like* in *Nicotiana benthamiana* leaf epidermal cells and onion epidermal cells. Epidermal cells of tobacco leaves and onion epidermal cells were infected with recombinant vector *35S::GhTIP1;1-like-GFP* and visualized by fluorescence microscopy. Onion epidermal cells were subjected to plasmolysis assay with 30% sucrose before taking pictures.

To determine the subcellular location of GhTIP1;1-like, a *35S::GhTIP1;1-like-GFP* fusion vector was constructed and transiently transformed into the inner epidermal cells of the onion and tobacco leaves. It was found that the green, fluorescent signal of the fusion protein (GhTIP1;1-like-GFP) was distributed throughout the membrane system of the inner epidermal cells (Figure 2).

### 2.4. Expression Pattern Analysis of GhTIP1;1-like under Abiotic Stresses

**Figure 3:**
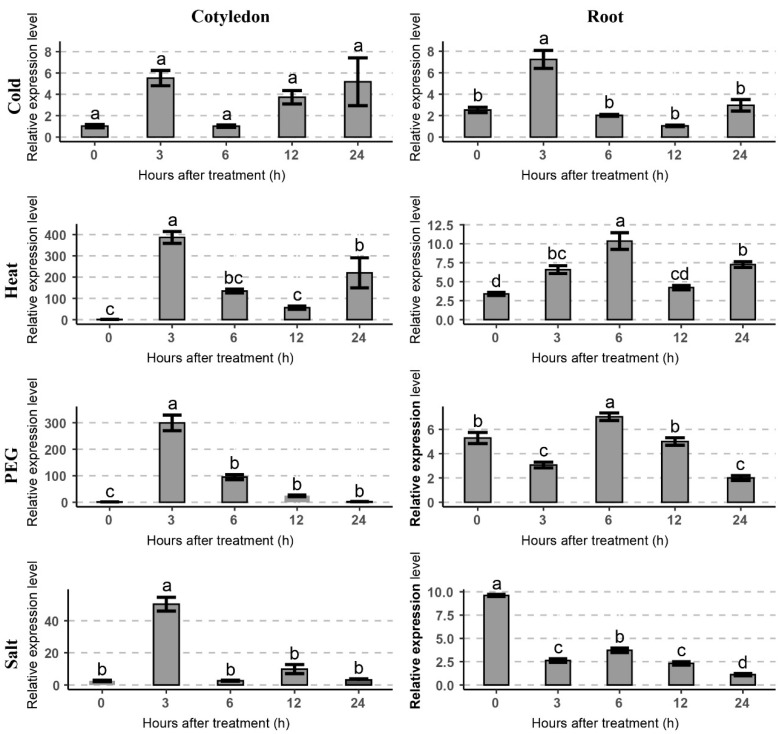
Expression patterns of *GhTIP1;1-like* in different tissues of 7-day-old cotton seedlings under different stress treatments. Expression levels of *GhTIP1;1-like* were determined from cotton cotyledons, and radicles under cold (4 °C), heat (42 °C), salt (300 mM NaCl), and dehydration (10% PEG6000) stresses using RT-qPCR analysis. Different letters indicate statistically significant differences (*p* < 0.05, Tukey’s test).

To investigate the effect of cold and other abiotic stresses on the expression level of *GhTIP1;1-like*, stress treatments were applied on young seedlings at the cotyledon stage (Figure 3). Expression of *GhTIP1;1-like* peaked at 3 h of cold treatment in cotyledons and roots. However, when compared with cold stress, the expression of *GhTIP1;1-like* could be strongly induced by heat, drought, and salt stress. In addition, we also found that *GhTIP1;1-like* was up-regulated in cotyledons after stress treatment, while the expression level in roots was lower.

**Figure 4:**
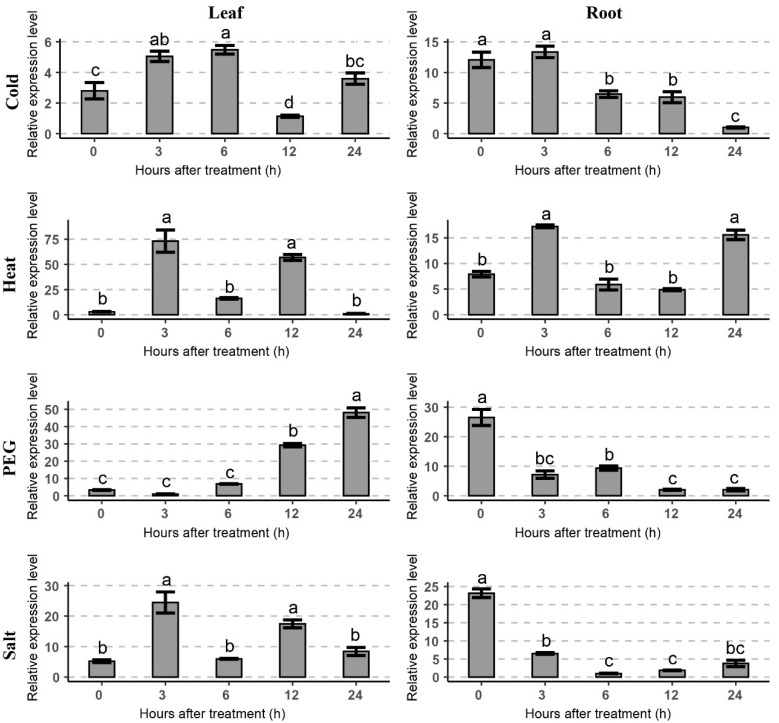
Expression patterns of *GhTIP1;1-like* in various tissues of 28-day-old cotton seedlings under different stress treatments. Expression levels of *GhTIP1;1-like* were determined from cotton leaf, and root under cold (4 °C), heat (42 °C), salt (300 mM NaCl), and dehydration (10% PEG6000) stresses using RT-qPCR analysis. Significant difference at *p* < 0.05 is indicated by different letters above the columns (Tukey’s test).

Previous studies have suggested that the cold tolerance of plants is different at different developmental stages [19,20]. Therefore, we analyzed the expression pattern of *GhTIP1;1-like* in cotton seedlings at true leaf stage in cold and other abiotic stresses by RT-qPCR (Figure 4). Before stress treatment, the expression level of *GhTIP1;1-like* in roots was significantly higher than that in leaves. Under cold treatment, the expression level of *GhTIP1;1-like* in roots and leaves gradually increased and then decreased, and it was higher in roots. In leaves, heat and salt stress treatments could induce the expression of *GhTIP1;1-like* to the peak at 3 h. However, when treated with PEG6000, the expression level of *GhTIP1;1-like* in the leaves gradually increased and peaked at 24 h of the treatment.

### 2.5. Chilling Tolerance Was Suppressed in GhTIP1;1-like-Silenced Cotton Seedlings

**Figure 5:**
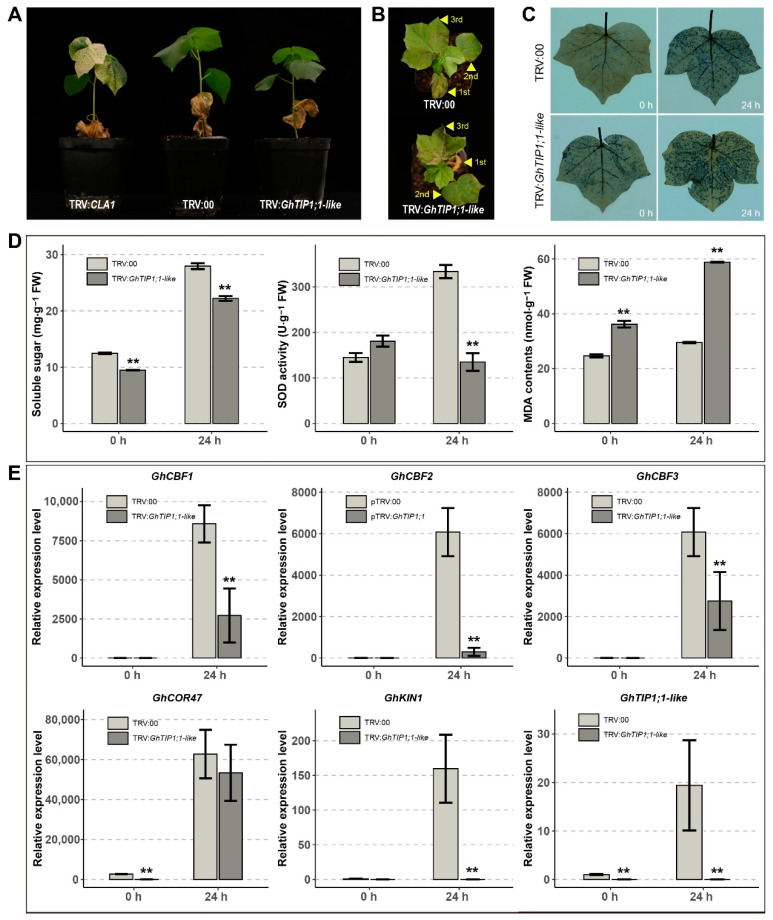
VIGS of *GhTIP1;1-like* gene in cotton. (**A**) The phenotype of cotton plants infiltrated with TRV:*CLA1*, TRV:00 and TRV:*GhTIP1;1-like*. (**B**) Cold stress recovery assay of the *GhTIP1;1-like*-silenced (TRV:*GhTIP1;1-like*) and control (TRV:00) plants after 3-days at 4 °C. Yellow triangles point to different true leaves. (**C**) NBT staining assay of the *GhTIP1;1-like* silenced and control plants in untreated (25 °C) and cold stress (4 °C) conditions. (**D**) Physiological indexes determination of *GhTIP1;1-like*-silenced and control plants in untreated and cold stress conditions. (**E**) Relative expression of cold-responsive genes in *GhTIP1;1-like*-silenced and control plants under cold stress conditions. Each experiment was repeated three times, with the bar representing the standard error (SE). Asterisks indicate a significant difference from the TRV:00 plants under the same cold condition at *p* < 0.01 as determined by a *t*-test.

To reveal the role of *GhTIP1;1-like* in cold tolerance of cotton, VIGS assay was performed to silence a 300 bp conserved sequence to inhibit the expression of *GhTIP1;1-like* in cotton. When the leaf of cotton seedlings infiltrated with TRV:*CLA1* showed white symptoms (Figure 5A), all seedlings infiltrated with TRV:00 or TRV:*GhTIP1;1-like* were tested with universal primers to obtain positive plants (Table S2). In the fourth week after infection, the positive plants were subjected to cold stress (4 °C), and RT-qPCR was performed to detect the gene silencing efficiency. When compared with the control (TRV:00), it was found that the expression level of *GhTIP1;1-like* in the silenced seedlings was significantly reduced after the cold treatment (24 h) (Figure 5E). The cellular O_2_^−^ level determined by nitroblue tetrazolium (NBT) staining was higher in the TRV:*GhTIP1;1-like* leaves than in the TRV:00 leaves after two days of cold treatment (Figure 5C). As we all know, physiological indicators are usually used to identify and evaluate the cold tolerance of plants, with soluble sugar (SS) and superoxide dismutase (SOD) as positive indicators. Malondialdehyde (MDA) is the product of lipid peroxidation, and its content negatively reflects the cold tolerance of plants. We found that after cold treatment, the SS content and SOD activity in the leaves of TRV:*GhTIP1;1-like* were significantly lower than the TRV:00, while the MDA content was higher than the control (Figure 5D). In addition, the expression levels of cold-responsive genes *GhCBF1*, *GhCBF2*, *GhCBF3* and *GhKIN1* were down-regulated in *GhTIP1;1-like*-silenced plants (Figure 5E). Phenotypic observation revealed that after 2 days of cold treatment, leaf damage of TRV:*GhTIP1;1-like* plants was more serious than TRV:00 (Figure 5B). These results indicated that silencing the *GhTIP1;1-like* gene resulted in decreased cold tolerance of cotton seedlings.

### 2.6. Identification of Transgenic Arabidopsis Lines

**Figure 6:**
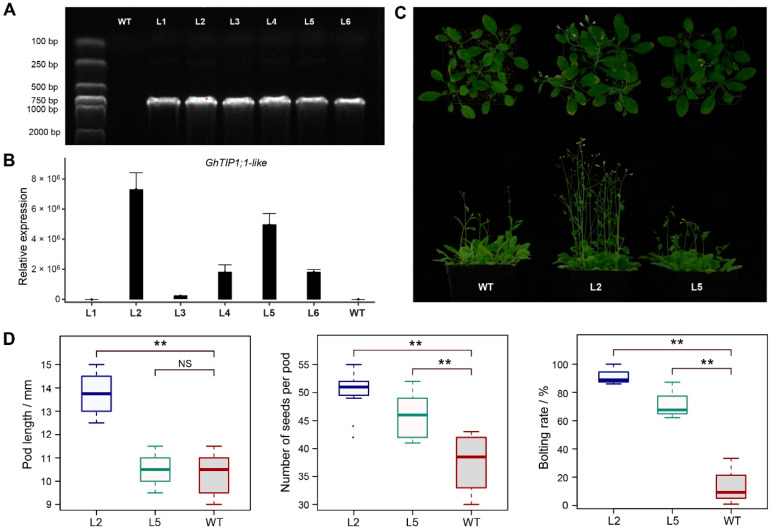
Overexpression of *GhTIP1;1-like* in *Arabidopsis*. (**A**) PCR analysis of transgenic plants. Lane M means DL2000 DNA marker; Lane WT means wild-type; Lanes L1-L6 means transgenic lines. (**B**) Expression levels of *GhTIP1;1-like* in Col-0 and transgenic lines determined by RT-qPCR. (**C**) Four-week-old WT and T2 transgenic plants. (**D**) Phenotypic characterization of WT and T2 transgenic plants. Three samples were measured in each experiment. Asterisks indicate a significant difference at *p* < 0.01 as determined by a Dunnett’s test, and NS indicate no significant difference at *p* < 0.05.

To investigate the role of *GhTIP1;1-like* in plant growth and cold response, transgenic *Arabidopsis* lines overexpressing *GhTIP1;1-like* under the control of the *CaMV 35S* promoter were generated. Six transgenic lines were obtained by kanamycin screening and PCR identification (Figure 6A). It was showed that *GhTIP1;1-like* was expressed in all transgenic lines but not in wild type (WT) plants (Figure 6B). Afterwards, we selected two transgenic lines with higher *GhTIP1;1-like* expression levels (L2 and L5) among the T1 lines for subsequent analysis (Figure 6B). The T3 homozygous lines were generated from L2 and L5 lines.

### 2.7. Overexpression of GhTIP1;1-like Promotes Precocious Bolting and Delays Senescence

It was known that overexpression of the tonoplast aquaporin gene could promote the growth of transgenic plants [14,21]. In our study, when compared with WT, the bolting of the L2 line was advanced (Figure 6C). Pod length and seed quantity per pod are important contributing factors to *Arabidopsis* yield. Further observation showed differences in pod length among different lines, and the pod length of L2 was significantly longer than that of WT. In contrast, the difference between L5 and WT was non-significant (Figure 6D). Seed number in the pods of each line was counted with a microscope, and statistical analysis showed that the seed number in the pods of L2 and L5 was significantly higher than that of WT (Figure 6D). In addition, the bolting rate of L2 and L5 was substantially higher than that of WT at the same time in the fourth week after sowing (Figure 6D).

**Figure 7:**
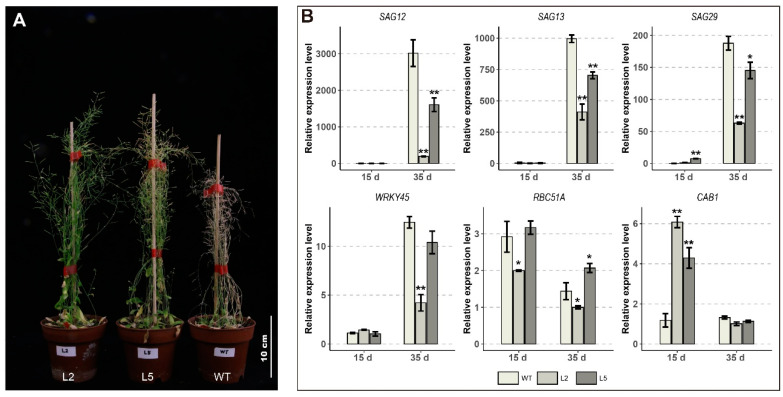
Phenotypic characteristics of transgenic plants at the senescence stage. (**A**) Differences in the senescence phenotype of WT and transgenic plants in the late growth period. (**B**) Relative expression of senescence-related genes in WT and transgenic plants. Each experiment was repeated three times, with the bar representing the standard error (SE). Asterisks indicate a significant difference from the WT plants at *p* < 0.05 or *p* < 0.01 as determined by Bonferroni’s Correction.

Furthermore, when compared with WT, the rosette leaves of the L2 line exhibited senescence symptoms. However, transgenic lines (L2 and L5) showed delayed senescence during the pod ripening stage (Figure 7A). In addition, we also determined the expression levels of senescence-related genes in different lines (Figure 7B). During plant senescence stage, the expression levels of *WRKY45* and *SAG* genes (*SAG12*, *SAG13* and *SAG29*) in transgenic plants were significantly lower than those of wild type, which was consistent with the results of previous studies [22,23]. These results suggest that *GhTIP1;1-like* may be involved in the regulation of plant reproductive growth and delaying senescence.

### 2.8. Overexpression of GhTIP1;1-like Enhances Cold Tolerance in Arabidopsis

**Figure 8:**
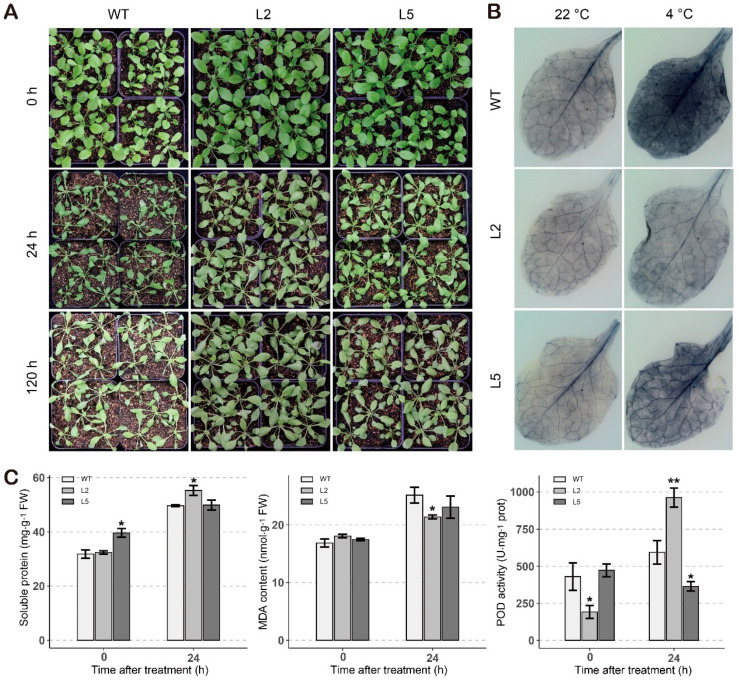
Overexpression of *GhTIP1;1-like* in *Arabidopsis* improves cold tolerance. (**A**) The effects of cold stress on transgenic plants and WT. (**B**) Histochemical staining assays were performed to detect O_2_^−^ by NBT staining. (**C**) Physiological indexes determination of transgenic lines and wild type plants in normal and cold environments. Each experiment was repeated three times, with the bar representing the standard error (SE). Asterisks indicate a significant difference from the WT plants at *p* < 0.05 or *p* < 0.01 as determined by Bonferroni’s Correction.

To further study the role of *GhTIP1;1-like* in cold tolerance, we performed cold treatment on the transgenic lines (L2 and L5) and WT. As shown in Figure 8A, the leaves of transgenic lines were still normal at 0 °C for 24 h after cold acclimation (at 4 °C for 24 h), while the WT plants showed dehydration and wilting symptoms. When treated at 0 °C for 120 h, symptoms of L2 and L5 plants were relatively normal, but the leaves of WT plant were severely dehydrated. It is known that plant tissues accumulate superoxide anions in adversity environments, which chemically react with nitrogen blue tetrazolium (NBT) to form formazan that is incompatible with water. Formazan is usually blue in colour and indicates the accumulation and distribution of O_2_^−^ in tissues. Before cold treatment, leaves of WT and transgenic plants were lightly stained, indicating that the content of O_2_^−^ was less accumulated. When treated at 4 °C for 24 h, the staining area of WT leaves was the largest, followed by L5 and L2 leaves (Figure 8B). After 24 h of cold stress, soluble proteins (SP) in L2 were significantly higher than that of WT, while there was no significant difference between L5 and WT (Figure 8C). After cold stress, the content of MDA in L2 was significantly lower than that of WT, while the activity of peroxidase (POD) was significantly increased (Figure 8C). These results indicate that overexpression of *GhTIP1;1-like* significantly enhances the cold tolerance of transgenic *Arabidopsis* plants.

### 2.9. Expression of Cold-Responsive Genes in Transgenic Arabidopsis

**Figure 9:**
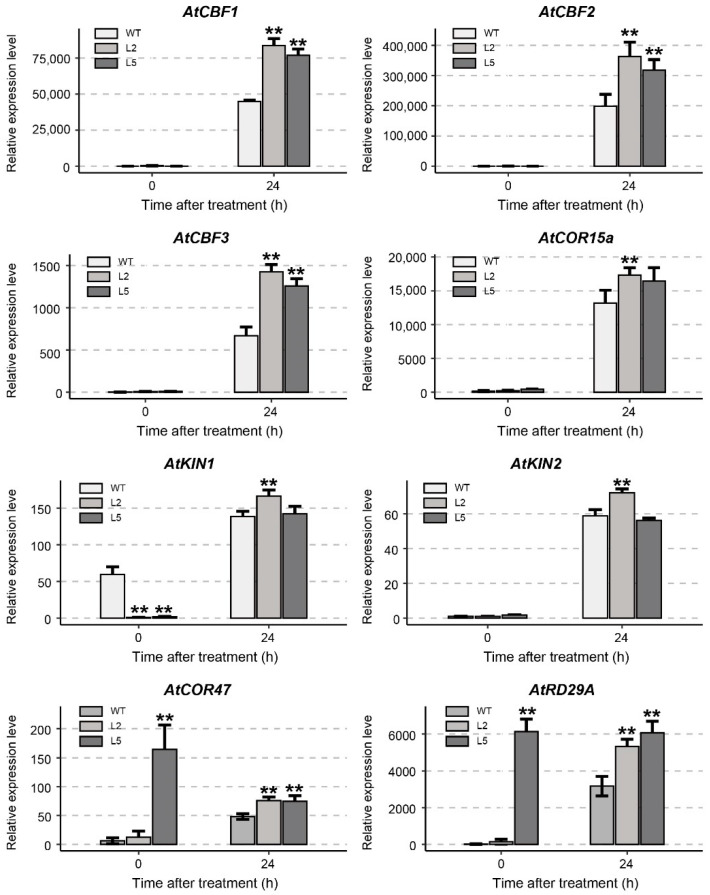
Expression analysis of cold-responsive genes in transgenic lines and WT plants before and after cold treatment. Each experiment was repeated three times, with the bar representing the standard error (SE). Asterisks indicate a significant difference from the WT plants under the same temperature condition at *p* < 0.01 as determined by a *t*-test.

Many studies have reported that the up-regulation of some genes involved in the cold response in *Arabidopsis thaliana* can enhance the cold tolerance of plants [24,25,26]. We performed RT-qPCR on eight cold-responsive genes in WT and transgenic lines (Figure 9). The *CBF* genes were induced by cold stress and the expression level was very low in normal temperature (22 °C). After 24 h of cold treatment, the expression levels of *AtCBF1*, *AtCBF2* and *AtCBF3* in transgenic lines were significantly higher than that of WT, and they were higher in L2 than that of L5. The expression levels of *AtCOR15a*, *AtKIN2* and *AtKIN1* were significantly up-regulated in transgenic plants after cold stress. Moreover, the expression levels of *AtCOR15a*, *AtKIN2*, *AtKIN1*, *AtCOR47* and *AtRD29A* were significantly up-regulated in transgenic plants after cold stress than that of WT. These results suggest that overexpression of *GhTIP1;1-like* in *Arabidopsis* can efficiently induce the expression of cold-responsive genes and enhance the cold tolerance of transgenic *Arabidopsis* plants.

### 2.10. Screening of Potential Proteins That Interact with GhTIP1;1-like

To explore the cold-responsive proteins interacting with GhTIP1;1-like, we constructed a yeast two-hybrid library. A total of 68 positive colonies were screened from the yeast library. Blast comparison was performed between sequencing results of the PCR products and ORF sequences of TM-1 from CottonFGD website (https://cottonfgd.org/, accessed on 3 February 2021), and 24 candidate genes encoding interacting proteins were obtained (Table S3). Among them, *BKI1* is associated with BR signal transduction, *PORA* encodes protochlorophyllide reductase, *BZIP10* is related to biotic stress response, *NAC062* and *COLD* are related to cold response, and *PIP2;7*, *TIP1;1* and *GhTIP1;1-like* are all members of the MIP superfamily.

**Figure 10:**
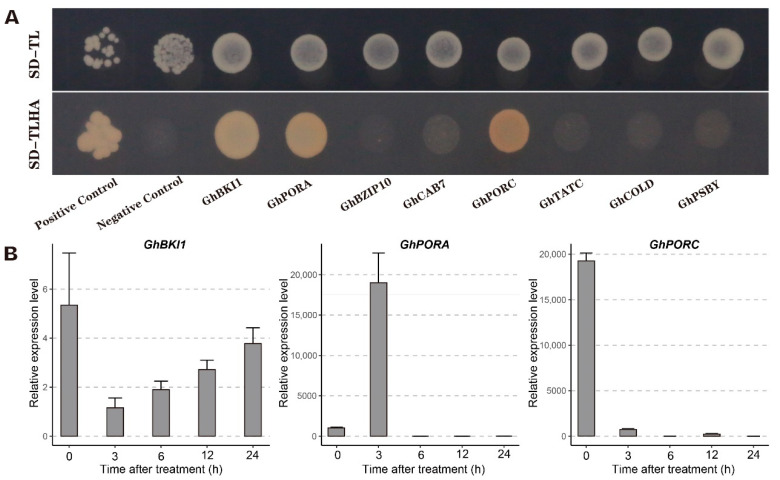
Interaction of GhTIP1;1-like with candidate proteins using a yeast two-hybrid assay. (**A**) Yeast two-hybrid analysis of the interaction between GhTIP1;1-like and other candidate proteins. The combination of the pGADT7-large T and pGBKT7-p53 plasmids was used as the positive control, and the combination of the pGADT7-large T and pGBKT7-laminC plasmids was used as a negative control. (**B**) Expression patterns of genes encoding potential proteins that interact with GhTIP1;1-like under cold treatments. Each experiment was repeated three times, with the bar representing the standard error (SE).

Eight candidate genes, *GhBKI1*, *GhPORA*, *GhBZIP10*, *GhCAB7*, *GhPORC*, *GhTATC*, *GhCOLD* and *GhPSBY* were cloned into pGADT7 vector and co-transformed with pGBKT7-*GhTIP1;1-like* integrated plasmid into Y2HGold yeast cells. The transformed cells were then cultured on SD/-Trp/-Leu (or SD-TL) and SD/-Trp/-Leu/-His/-Ade (or SD-TLHA) solid plates. All the colonies grew normally on SD/-Trp/-Leu plates, but only the positive control and yeasts co-transformed pGBKT7-*GhTIP1;1-like* with pGADT7-*GhBKI1*, pGADT7-*GhPORA*, or pGADT7-*GhPORC* could grow on SD-TLHA plate, as shown in Figure 10A. These results indicate that GhTIP1;1-like can interact with GhBKI1, GhPORA or GhPORC, but cannot interact with GhBZIP10, GhCAB7, GhTATC, GhCOLD or GhPSBY. To explore whether the three genes really respond to cold stress, we analyzed the expression levels of them. With the extension of cold stress, the expression level of *GhBKI1* gradually increased (Figure 10B). Interestingly, we found that *GhPORA* was strongly induced by cold stress and the expression level peaked at 3 h of cold treatment. However, the expression of *GhPORC* was suppressed by cold stress, suggesting that GhPORA may enhance the cold tolerance of cotton by interacting with GhTIP1;1-like.

### 2.11. Promoter Analysis of GhTIP1;1-like

**Figure 11:**
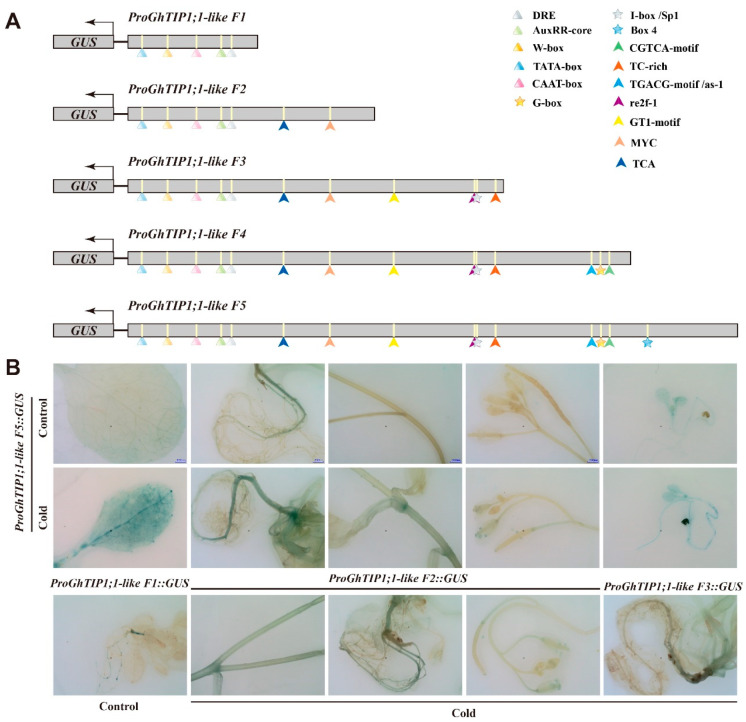
Promoter deletion analysis of *GhTIP1;1-like* under different temperature conditions. (**A**) Schematic representation of *ProGhTIP1;1-like::GUS* constructs. Different symbols indicate different motifs of interest. (**B**) Histochemical GUS staining of transgenic *Arabidopsis* with *ProGhTIP1;1-like F5*, *ProGhTIP1;1-like F3*, *ProGhTIP1;1-like F2*, and *ProGhTIP1;1-like F1* under normal-temperature (22 °C) and cold conditions (4 °C).

The *cis*-elements in the *GhTIP1;1-like* promoter was predicted using the PlantCARE program. The results showed that the response of the elements to light (e.g., G-box, GT1-motif, ATCT-motif, Box 4. GATA-motif and I-box), plant hormone (e.g., ERE, AUXRR-core, and ABRE), or stress (e.g., DRE-core, ARE, MYC, STRE, and W-box) were present in the promoter (Table S4). Transcription start site (TSS) was predicted by the Softberry program to be at −78 bp upstream of the initiator codon ATG. According to the detailed location of predicted *cis*-elements and TSS, a 2554 bp sequence named *ProGhTIP1;1-like* was cloned as the promoter of *GhTIP1;1-like* from −33 bp upstream of ATG to the 5′-region. The promoter was properly truncated into five fragments and introduced into pBI121 plasmid to construct fusion vectors with the GUS gene, respectively. The construction products schemed as shown in Figure 11A, full-length pBI121-*ProGhTIP1;1-like* was named *ProGhTIP1;1-like F5* and the other vectors were named *ProGhTIP1;1-like F4*, *ProGhTIP1;1-like F3*, *ProGhTIP1;1-like F2*, and *ProGhTIP1;1-like F1*, respectively. The truncated vectors were transformed into *Arabidopsis*, for which the GUS staining results in control (22 °C) and cold (4 °C) environments were shown in Figure 11B. Transgenic *Arabidopsis* seedlings integrated with *ProGhTIP1;1-like F5::GUS* were partially stained in the leaves and roots at 22 °C. After cold treatment, GUS activity was additionally detected in stems and anthers, and GUS activity in leaves and roots was enhanced compared with the control. It was also revealed that the roots of transgenic plants carrying *ProGhTIP1;1-like F1::GUS* were partially stained blue at 22 °C. After cold treatment, the roots, stems and anthers of *ProGhTIP1;1-like F2::GUS* were stained, and the root staining was similar to *ProGhTIP1;1-like F1::GUS*, indicating that *ProGhTIP1;1 F2* had the activity of cold response. When comparing *ProGhTIP1;1-like F2* with *ProGhTIP1;1-like F1*, we found an MYC motif that included in the extra cis-acting elements. It has been reported that MYC is an essential *cis*-element response to cold stress, suggesting that the motif may enhance the response of *ProGhTIP1;1-like F2* to cold stress [27].

## 3. Discussion

### 3.1. The Expression of GhTIP1;1-like Is Affected by Temperature, Drought and Salt Stress

In a previous study, a gene annotated as aquaporin was found in a significant module in the transcriptome database [16]. The protein sequence analysis revealed that it was homologous to AtTIP1;1. However, functional characterization of the tonoplast aquaporin in cotton remains unclear. It has been found that overexpression of the ginseng aquaporin gene *PgTIP1;1* in *Arabidopsis* could affect the response and resistance of transgenic plants to abiotic stresses, such as salt, drought and cold [14]. Yin et al. found that different stresses such as low temperature, salt, mannitol, salicylic acid, ABA and *Phytophthora capsicum* infection could induce the up-regulation of *CaPIP1;1* in pepper [28]. Under high salt and mannitol treatments, the expression level of *CaTIP1;1* gene in the radicle of transgenic plants was higher than that of wild type, and the plant growth was more vigorous [29]. Moreover, overexpression of the pepper gene *CaTIP1;1* in tobacco could enhance the antioxidant enzyme activities under osmotic stress, and the expression level of genes related to reactive oxygen species (ROS) regulation was up-regulated [29]. In our study, the cotton *GhTIP1:1-like* gene could respond to cold and other abiotic stresses, such as heat, salt and drought. It was revealed that the *GhTIP1;1-like* gene was up-regulated in the cotyledons after 3 h of cold treatment, and the gene was hypersensitive to heat, drought and salt stresses (Figure 3). Additionally, the expression of *GhTIP1;1-like* was also induced by various abiotic stresses in true leaves (Figure 4). However, when *Festuca arundinacea* was subjected to cold stress, the expression of *TIP1;1* was down-regulated [30]. It has been confirmed that the rice gene *OsTIP*s could respond to drought and salt stress [31]. Although the tonoplast aquaporin gene in plants has not been reported to respond to heat stress, the *TIP1* identified in yeast could be induced by cold and heat stress [32]. Expression analysis revealed that *GhTIP1;1-like* is hypersensitive to heat stress, suggesting that it may play an important role in heat response

### 3.2. Overexpression of GhTIP1;1-like Accelerates Plant Growth and Affects Plant Senescence

In plants, AtTIP1 is the first protein identified to function as a water transport channel [33]. In addition to allowing fast and passive diffusion of water across vacuole membrane, TIPs also facilitate the transmembrane transport of glycerol, urea, H_2_O_2_, NH_4_^+^/NH_3_, methylammonium and formamide [34,35,36,37,38]. Bienert et al. found that *AtTIP1;1* and *AtTIP1;2* could promote the diffusion of H_2_O_2_ across the membrane. In plant roots, the expression level of *TIP1* is higher in the cells surrounding the developing vascular tissue, and high levels of ROS were also detected in this tissue [39,40]. Researchers speculate that *TIP1;1* may play a role in regulating H_2_O_2_ levels during the formation of the xylem. In addition, the high expression level of *ZmTIP1* is conducive to the transport of water across the membrane. In plants, leaf senescence is related to differences of ROS accumulation in cells and the regulation of antioxidant systems [41]. It is believed that plant senescence is associated with lipid peroxidation, the increase of biomembrane permeability, and the decrease of catalase activity [42]. We suspect that *GhTIP1;1-like* may promote the transmembrane transport of H_2_O_2_, and changes the homeostasis of cellular ROS, which in turn affects leaf senescence. It was reported that suppression of *AtTIP1* expression induced premature senescence and death of transgenic plants [13]. Further analysis of *TIP1;1* gene in the regulation of ROS homeostasis suggests that loss of the *TIP1;1* function will lead to the accumulation of H_2_O_2_ in the cytosol, which in turn contributed to cell death and leaf senescence. In our study, overexpression of *GhTIP1;1-like* resulted in early senescence symptoms of rosette leaves but delayed the later senescence process of the transgenic plant, suggesting that the regulation mechanism of *GhTIP1;1-like* in plant senescence may be more complicated. In our study, overexpression of *GhTIP1;1-like* promoted *Arabidopsis* bolting and accelerated plant growth.

### 3.3. Overexpression of GhTIP1;1-like Enhances Cold Tolerance of Plants

A previous study has found that overexpression of ginseng *PgTIP1;1* gene in *Arabidopsis thaliana* confers salt tolerance to plants, and the expression levels of stress-related genes such as *CBF3*, *ZAT12*, *MYB15*, *COR47*, *ICE1* and *LTI78* were up-regulated in transgenic plants [21]. It was not reported whether *PgTIP1;1* affected the cold tolerance of plants, but it is well known that these genes (*CBF3*, *ZAT12*, *MYB15*, *COR47*, *ICE1* and *LTI78*) are all related to cold tolerance. Therefore, the *TIP1;1* gene may confer cold tolerance to transgenic plants. In this study, both virus-induced gene silencing and overexpression of *GhTIP1;1-like* revealed that the gene contributed to the cold tolerance of plants. For example, superoxide anion content was less accumulated in transgenic *Arabidopsis* plants compared with WT after cold stress (Figure 8B), while SP content and POD activity were significantly increased (L2). Additionally, cold-responsive or -resistant genes were significantly up-regulated after cold treatment when compared with wild-type. Yeast two-hybrid assay showed that the GhTIP1;1-like protein could interact with GhBKI1 and two protochlorophyllide reductases (GhPORA and GhPORC). In our study, the expression of *GhPORA* was induced by cold stress, while *GhPORC* was inhibited (Figure 10B). Therefore, further research on the interaction between GhTIP1;1-like and candidate proteins may be of great significance for analyzing the cold response mechanism of cotton seedlings.

## 4. Materials and Methods

### 4.1. Plant Material and Treatments

The seeds of wild-type *Arabidopsis thaliana* (Col-0) provided by Shanghai Weidi Biotechnology Co., Ltd. (Shanghai, China) were sterilized with 1% sodium hypochlorite solution and 75% alcohol solution and then sprinkled on 1/2 MS solid medium for vernalization. The germinating seeds were routinely grown in a growth chamber (Saifu, Ningbo, China) at 22 °C with a photoperiod of 16 h-light/8 h-dark. After three weeks, the wild-type and transgenic *Arabidopsis* seedlings were treated with 4 °C for 24 h and then transferred to 0 °C. Leaves were collected before 4 °C and after 0 °C treatment for physiological indicators determination and RT-qPCR. At the same time, phenotypes of seedlings were observed and photographed during cold treatment.

The seeds of the upland cotton cultivar YM21 screened from a previous study were sown in different pots. When the cotyledons were to be flattened, the seedlings were pulled out and sprayed with water to remove the soil from the radicle and then transferred to Hoagland nutrient solution for hydroponic culture. The seedlings at the cotyledon stage and two-leaf stage were treated with abiotic stresses, and samples were collected. Before treatment, the seedlings were transferred into a conical flask containing 100 mL of different solutions. The seedlings cultivated with Hoagland nutrient solution in a plant incubator were used for cold or heat treatment. The 300 mM NaCl (Solarbio, Beijing, China) solution and 10% PEG6000 (Solarbio, Beijing, China) solution were used to simulate salt stress and drought stress, respectively. The leaves and roots were collected at 0, 3, 6, 12 and 24 h under different abiotic stresses, and the samples were quick-frozen in liquid nitrogen. To analyze the tissue-specific expression level of *GhTIP1;1-like* in TM-1, roots, stems and leaves were collected from seedlings, and buds, sepals, petals, stamens and fiber samples.

### 4.2. Isolation and Cloning of the ORF and Promoter of GhTIP1;1-like

Total RNA was extracted from the cotyledons of cold-tolerant cotton cultivar H559 at 3 h of cold stress. The extraction of RNA and the synthesis of cDNA were performed as previously described methods [16]. The Oligo 7.0 software was used to design the primer sequences of the genes, and the full-length coding sequences were amplified by PCR. Geneious v4.8.4 software was used to convert gene coding sequences into amino acid sequences, and BLASTp program was used to align the protein sequences to the NCBI protein database. Multiple protein sequences were downloaded and performed alignment with the help of DNAMAN 6.0 (Lynnon Biosoft, San Ramon, CA, USA). The phylogenetic tree was carried out using MEGA 6.0. The ProtParam tool of the online website ExPASy (https://web.expasy.org, accessed on 4 September 2020) was used to predict protein molecular weight and pI. To isolate the promoter fragment of *GhTIP1;1-like*, gDNA of *Gossypium hirsutum* cv. TM-1 was extracted. The online program PlantCARE (http://bioinformatics.psb.ugent.be/webtools/plantcare/html/, accessed on 4 September 2020) was used to analyze *cis*-acting elements in the promoter of *GhTIP1;1-like* [43]. Afterwards, the promoter region was divided into five overlapping and truncated fragments, and the sequence of promoter primers was shown in Table S2. The cloning method of truncated promoter fragments was previously described [44].

### 4.3. Plasmid Construction

To generate the overexpression plasmid, the *GhTIP1;1-like* coding sequence was cloned into the pBI121 vector under the control of the *CaMV 35S* promoter. To examine the promoter activity of *GhTIP1;1-like*, a 2587 bp promoter fragment was inserted into the HindIII and XbaI sites of the pBI121 vector to generate *ProGhTIP1;1-like::GUS* plasmids. To silence the cotton *GhTIP1;1-like* gene, a 300 bp conservative fragment in the coding sequence of the gene was obtained with the help of the online website (https://vigs.solgenomics.net, accessed on 22 October 2020), and the fragment was cloned into the TRV:00 plasmid. To generate the subcellular localization construct, the *GhTIP1;1-like* coding region was cloned into the pBI121-GFP vector to express GhTIP1;1-like::GFP fusion protein under the control of the *CaMV 35S* promoter. To express GhTIP1;1-like protein in yeast, the ORF was cloned into pGBKT7 vector to generate the *pGBKT7::GhTIP1;1-like* fusion construct. All fusion vectors were first transformed into competent *E. coli* strain *Trelief*^TM^ 5α. The fidelity of the constructs was confirmed by sequencing.

### 4.4. Plant Transformation

The recombinant plasmid *35S::GhTIP1;1-like*, *ProGhTIP1;1-like::GUS*, *35S::GhTIP1;1-like::GFP* and TRV:*GhTIP1;1-like* were transformed into *Agrobacterium tumefaciens* strain GV3101 or LBA4404 (Weidi, Shanghai, China). The GV3101 cells harbouring the fusion constructs *35S::GhTIP1;1-like* and *ProGhTIP1;1-like::GUS* were transformed into Col-0 *Arabidopsis thaliana* plants via the floral dip method [45]. Transgenic *Arabidopsis* seedlings were screened using 1/2 MS medium containing 50 mg·L^−1^ kanamycin (Solarbio, Beijing, China) and further confirmed by PCR and RT–qPCR. The *Agrobacterium* strains containing TRV1 and those that were containing TRV:*GhTIP1;1-like* or TRV:00 were mixed in equal volume and infiltrated into the cotyledons of seven-day-old seedlings of H559 by syringe infiltration to generate the control (TRV:00) and *GhTIP1;1-like*-silenced (TRV:*GhTIP1;1-like*) cotton plants [46]. TRV:*CLA1* was used as a positive control as previously described. The *Agrobacterium* strain GV3101 carrying the recombinant vector *35S::GhTIP1;1-like::GFP* was used to infect onion epidermal cells and tobacco leaves for subcellular localization as previously described [47].

### 4.5. Y2H Library Assay

Young cotton cotyledons were cultivars H559 and YM21 were treated at 4 °C and collected at 0, 3 and 6 h, and subjected to total RNA extraction and mRNA isolation. The construction of the yeast two-hybrid library was completed by the service of Shanghai OE Biotech. Co., Ltd. (Shanghai, China). The pGBKT7-*GhTIP1;1-like* fusion plasmid was transformed into Y2HGold competent cells alone to obtain positive strains for Y2H library screening with the yeast mating method. Yeast colonies with diameters greater than 2 mm were identified by PCR and sequencing. The sequences were aligned to the reference genome of *Gossypium hirsutum* (https://cottonfgd.org, accessed on 3 February 2021). The ORFs encoded potential interacting proteins were cloned into the pGADT7 vector. All primer sequences of candidate interacting genes obtained by Y2H were shown in Table S5. Each recombinant construct was co-transformed into the yeast strain Y2HGold with the pGBKT7-*GhTIP1;1-like* plasmid. Yeast transformants were tested on both SD/-Trp/-Leu and SD/-Trp/-Leu/-His/-Ade medium at 30 °C for 5 days. The pGBKT7-p53 and pGADT7-largeT plasmids were used as positive controls, and pGBKT7-laminC and pGADT7-largeT were used as negative controls.

### 4.6. Histochemical Staining Assays

Transgenic *Arabidopsis* plants harbouring *ProGhTIP1;1-like F1::GUS*, *ProGhTIP1;1-like F2::GUS*, *ProGhTIP1;1-like F3::GUS*, *ProGhTIP1;1-like F4::GUS*, and *ProGhTIP1;1-like F5::GUS* fragments in the genome were generated via the floral dip method, and T2 progenies were used to analyzing promoter activity. GUS staining was performed as previously described [48]. Treated samples were immersed in GUS histochemical staining buffers (Huayueyang Biotechnology Co., Ltd., Beijing, China) and incubated overnight at 37 °C in the tin foil. After staining, the samples were decolourized in 75% ethanol until the colour of the negative control turned white. Eventually, GUS activity was examined based on the presence of blue.

Accumulation of ROS content in transgenic *Arabidopsis* plants or *GhTIP1;1-like*- silenced cotton seedlings was determined by assessing the levels of superoxide anion (O_2_^−^) via NBT staining according to previous descriptions [49].

### 4.7. Determination of Physiological Indexes

Activities of POD, SOD and the contents of MDA, SS and SP were determined before (0 h) and after (24 h) cold treatments. SOD and POD activities were measured according to the manufacturer’s instruction (Comin, Suzhou, China). MDA, SS and SP were determined by MDA, SS and SP kits (Comin, Suzhou, China), respectively.

### 4.8. PCR and RT-qPCR

The genomic DNA of cotton and *Arabidopsis thaliana* were extracted by the improved CTAB method and TPS method, respectively. Oligo 7.0 software was used to design gene primers for PCR or RT–qPCR. The primer sequences are shown in Tables S2 and S6. The Phanta^®^ Max Super-Fidelity DNA Polymerase product (P505) was used to clone cotton genes. V2 × Taq Plus Master MixII high-fidelity enzyme was used for PCR detection of transgenic *Arabidopsis* plants. FastPure^®^ Gel DNA Extraction Mini Kit was used to recover target fragments from agarose gel. All of these test products were provided by Vazyme Biotech Co., Ltd. (Nanjing, China). Total RNA extraction and cDNA synthesis were carried out according to the previous description [16]. The cDNA was used as a template to perform RT-qPCR on the ABI7500 instrument (Applied Biosystems, Waltham, MA, USA) for gene expression analysis, and three biological replicates were set. The RT–qPCR program was performed as follows: 95 °C, 10 s; 95 °C, 15 s and 60 °C, 60 s for 40 cycles; then a melting curve. *GhACTIN7* was used as an internal reference gene for expression pattern analysis in cotton [50]. The relative expression of genes was calculated using the 2^−ΔΔCt^ method [51].

### 4.9. Statistical Analysis

The statistical software SPSS 23.0 and R v3.6.2 software were used for data analysis and drawing, respectively. Tukey’s test, Dunnett’s test, Bonferroni’s Correction and Student’s *t*-test were used to calculate statistical significance, and the significance level and extremely significant level were set at *p* < 0.05 and *p* < 0.01, respectively. Tukey’s test was used for statistical analysis of the data for expression patterns of *GhTIP1;1-like* in cotton seedlings. Dunnett’s test was used for statistical analysis of the data for pod length, seed number per pod and the bolting rate. Bonferroni’s Correction and Student’s *t*-test were used for statistical analysis of the data for physiological indexes and gene expression levels in transgenic plants and wild-type (cotton or *Arabidopsis*).

## 5. Conclusions

In our study, gDNA and cDNA of *GhTIP1;1-like* gene was successfully cloned from upland cotton. Tissue-specific expression pattern analysis showed that the expression of *GhTIP1;1-like* was induced not only by cold stress but also by heat, drought and salt stresses. Multiple sequence analysis revealed that GhTIP1;1-like was homologous with TIP1 protein from other species. An MYC *cis*-element related to cold response was found in the *GhTIP1;1-like* promoter sequence and GUS staining showed that the promoter activity of the *GhTIP1;1-like* was enhanced by cold stress. In addition, overexpression of *GhTIP1;1-like* in *Arabidopsis* could delay senescence and enhance cold tolerance of transgenic plants. However, the cold tolerance of cotton plants was decreased by VIGS of *GhTIP1;1-like*. Physiological indexes revealed that the transgenic lines were more tolerant to cold stress than the wild type. The expression level of cold-responsive genes in transgenic plants was significantly up-regulated. Our study provides an essential basis for further research on the positive role of aquaporin genes in plant cold tolerance.

## Data Availability

The data presented in this study are available in the article and Supplementary Files.

## Supplementary Materials

The following supporting information can be downloaded at: www.mdpi.com/article/10.3390/ijms23031361/s1.
